# Bottom-up synthetic ecology study of microbial consortia to enhance lignocellulose bioconversion

**DOI:** 10.1186/s13068-022-02113-1

**Published:** 2022-02-07

**Authors:** Lu Lin

**Affiliations:** Institute of Marine Science and Technology, Shandong University, Qingdao, 266237 Shandong China

**Keywords:** Lignocellulose bioconversion, Microbial community, Microbial interactions, Synthetic ecology

## Abstract

Lignocellulose is the most abundant organic carbon polymer on the earth. Its decomposition and conversion greatly impact the global carbon cycle. Furthermore, it provides feedstock for sustainable fuel and other value-added products. However, it continues to be underutilized, due to its highly recalcitrant and heterogeneric structure. Microorganisms, which have evolved versatile pathways to convert lignocellulose, undoubtedly are at the heart of lignocellulose conversion. Numerous studies that have reported successful metabolic engineering of individual strains to improve biological lignin valorization. Meanwhile, the bottleneck of single strain modification is becoming increasingly urgent in the conversion of complex substrates. Alternatively, increased attention has been paid to microbial consortia, as they show advantages over pure cultures, e.g., high efficiency and robustness. Here, we first review recent developments in microbial communities for lignocellulose bioconversion. Furthermore, the emerging area of synthetic ecology, which is an integration of synthetic biology, ecology, and computational biology, provides an opportunity for the bottom-up construction of microbial consortia. Then, we review different modes of microbial interaction and their molecular mechanisms, and discuss considerations of how to employ these interactions to construct synthetic consortia via synthetic ecology, as well as highlight emerging trends in engineering microbial communities for lignocellulose bioconversion.

## Background

**Fig. 1:**
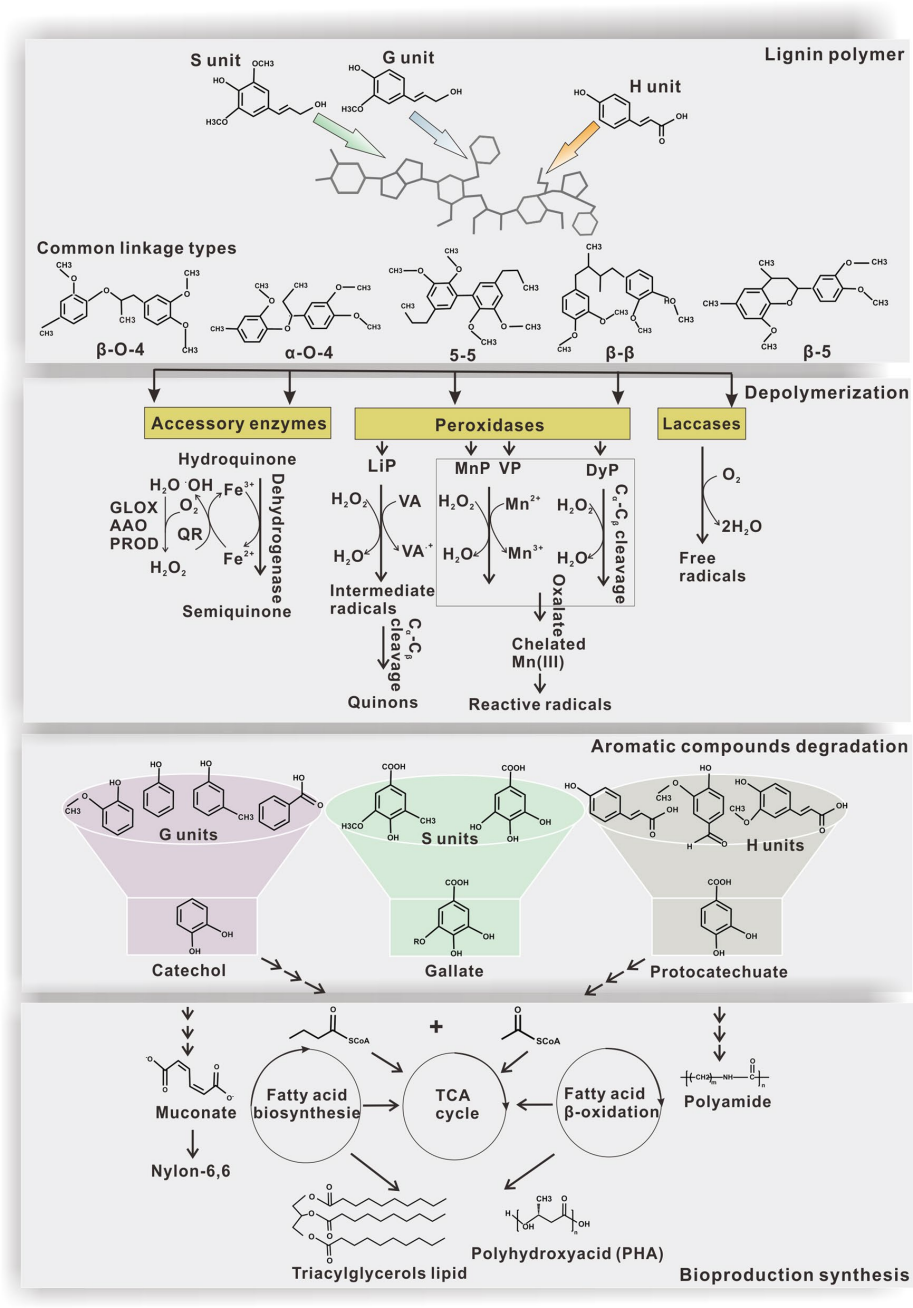
The structure of lignin and microbial mediated lignin bioconversion. Lignin is the most recalcitrant component of lignocellulose. It’s composed of G, S, and H phenylpropanoid constitutes, which are linked by five linkage types. These common linkages are cleaved by lignolytic enzymes (e.g., laccases, peroxidases, and redox accessory enzymes) for lignin depolymerization. Subsequently, aromatic compounds from G-, H-, and S-type lignin are degraded via microbial funnel pathways to generate the key intermediate aromatic compounds protocatechuate, catechol, and gallate. These intermediates are further catabolized to synthesize value-added bioproducts e.g., polyamide and nylon. Alternatively, they enter TCA cycle and fatty acid metabolism for PHA or lipid synthesis

Lignocellulose, which is chiefly composed of cellulose, hemicellulose and lignin, is the most abundant organic carbon polymer in the biosphere. Take lignin for instance, with an estimated 300 billion tons present globally and an annual increase of ~ 20 billion tons [1]. Its decomposition and conversion have a significant impact on the global carbon cycle. Moreover, this abundance enables its use as a feedstock for sustainable fuel and chemical production (e.g., ethanol, nylon, lipid and polyhydroxyalkanoate (PHA)), addressing energy and environmental concerns (Fig. 1) [1–3]. However, lignocellulose has remained the underutilized renewable biomass due to its complex chemical structure. This is especially true for lignin. Unlike cellulose and hemicellulose, which consists of carbohydrate monomers, lignin is composed of three phenylpropanoid units, which are linked by various, strong carbon–carbon and ether bonds, to form natural barriers against decay and give plants their rigidity and structure (Fig. 1) [1]. So far, lignin is removed during the pretreatment process at lignocellulosic biorefineries and used as fuel to supply heat and power through burning. Thus, lignin valorization not only provides opportunity to produce value-added chemicals and fuels, but also improves the efficiency of the current lignocellulosic biorefineries.

Microorganisms, which are present in nearly all habitats on the Earth, have evolved different, yet complementary, mechanisms to degrade these polymers. Therefore, they provide green and energy-efficient routes for lignocellulose bioconversion [4, 5].

i) Various enzymes have been identified as lignocellulose biocatalysts. Fungi and bacteria release cohorts of carbohydrate-active enzymes (CAZymes, e.g., glycoside hydrolases (GHs)) to synergistically hydrolyze (hemi) cellulose. For instance, aerobic fungi secrete ample and distinct cellulases, as noncomplexed cellulase mixtures, to hydrolyze cellulose, while some anaerobic bacteria, e.g., *Clostridium* strains, produce cell-bound cellulosomes for efficient cellulose hydrolysis [6]. Moreover, microorganisms have diverse sets of oxidoreductase enzymes for lignin oxidization [4, 7–9]. Recently, bacterial enzymes have been reported to play a key role in lignin oxidization [9, 10]. Our lab indicated that the dye-decolorizing peroxidases (DypBs) in *Pseudomonas putida* A514 showed a unique Mn^2+^ independent lignin depolymerization activity and exhibited synergistic lignin degradation with *P. putida* cells [11]. Furthermore, we bio-designed two secretory apparatuses for enhanced extracellular laccase expression to promote lignin degradation [12].
ii) Highly versatile metabolic pathways contribute to achieve lignocellulose conversion. With the development of systems biology and synthetic biology, we and others have developed various genetic engineering strategies to engineer metabolic pathways for enhancement of lignocellulose bioconversion [4, 13–15]. Shaw et al. deleted genes involved in organic acid formation in *Thermoanaerobacterium saccharolyticum* to significantly improve cellulosic ethanol production [16]. Jin et al. introduced a rationally designed hydroxylase system in *Rhodococcus opacus* to accumulate gallate, used o-demethylation systems to convert multiple lignin-derived methoxy aromatics to gallate, and engage an aryl side-chain oxidase to broaden the substrate spectrum [17]. As a result, they greatly improved lignin to gallate conversion. We also applied systems biology to guide design of lignin-to-PHA bioconversion in *P. putida* [18] and then, developed a strategy to simultaneously improve cell growth and PHA production in *P. putida* from a lignin derivative [19]. Moreover, for enhancement of ferulic acid (a lignin derivative)-to-PHA bioconversion, we developed a CRISPR/Cas9n-based genome editing tool to metabolically engineer *P. putida* [20]. These studies laid an essential foundation in understanding the molecular mechanisms of lignocellulose bioconversion and further engineering microorganisms for enhancement of lignocellulose bioprocess.

With numerous studies reporting successful metabolic engineering of individual strains, the bottleneck of single strain modification is becoming more urgent in the conversion of complex substrates, because lignocellulose conversion involves multiple enzymes and pathways that generally do not exist within, nor can be feasibly introduced into, a single strain. In natural environments, microorganisms form communities, where each member executes specialized sub-functions to synergistically perform the complete lignocellulose bioconversion [21]. Moreover, in contrast to pure cultures, microbial communities exhibit the property of resilience, which can increase resistance to environmental perturbations [22, 23]. Undoubtedly, natural microbial communities can provide clues to expand microbial engineering to mixed consortia. This review discusses current developments in microbial consortia for lignocellulose degradation, microbial interaction modes and molecular mechanisms, considerations of how to construct synthetic consortia, as well as the trends in designing and engineering synthetic microbial communities for lignocellulose bioconversion.

## Development of microbial consortia for lignocellulose degradation

**Fig. 2:**
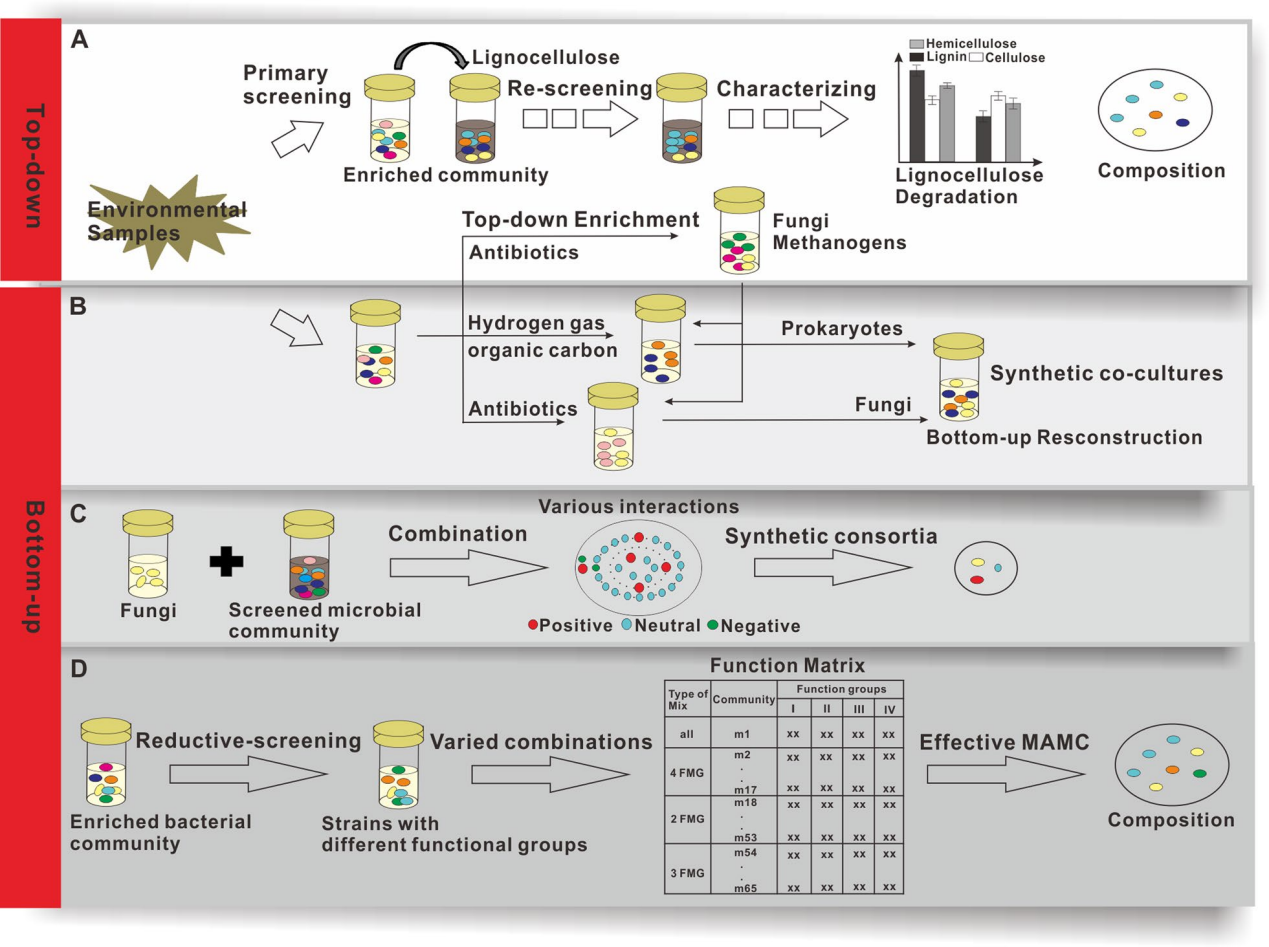
The current strategies to develop microbial consortia for lignocellulose bioconversion. **A** “Top-down” selection and enrichment strategy. After manipulation via primary screening, re-screening, and identification, a group of microbial consortia are developed. **B**–**D** “Bottom-up” reconstruction of co-cultures. **B** “bottom-up” co-cultures are constructed based on potential interactions predicted by the genomic sequencing of “top-down” enriched consortia [25]. **C** A combination strategy is used to develop fungal-bacterial microbial consortia, based on as many strains as possible with as few experiments as possible [40]. **D** A reductive-screening approach with ecological strategies is developed to acquire an effective lignocellulose-degrading minimally active microbial consortium [37]. FMG: functional metabolic groups

As stated previously, consortium-based approaches are a promising avenue for lignocellulose bioconversion [24, 25]. To date, “top-down” enrichment and “bottom-up” synthetic communities are the two major routes in developing microbial consortia for lignocellulose degradation (Fig. 2).

### “Top-down” selection and enrichment

“Top-down” selection and enrichment (e.g., dilution-to-stimulation method) is the most common strategy to obtain microbial consortia with lignocellulose degradation properties from natural environments [26, 27]. In this route, microbial samples from different environmental sources are collected, screened, and enriched after generations of subculture. Through primary screening and rescreening, the composition of the microbial communities gradually changes to adapt to the current conditions. Finally, stabilized microbial communities are generated, where members synergistically interact to degrade lignocellulose (Fig. 2A). Fang et al. employed this method to develop the microbial consortium DM-1 from tree trimmings [28]. DM-1 included *Mesorhizobium*, *Cellulosimicrobium*, *Pandoraea*, *Achromobacter*, and *Stenotrophomones* as the predominant genera and efficiently degrades lignin (28.7%) and cellulose (10.2%). Cortes-Tolalpa cultured a salt marsh soil microbiome in six sequential cycles on fresh wheat straw and four cycles on pre-digested wheat straw. Consequently, a consortium, which was selected on the latter, highly-recalcitrant substrate, improved cellulose (64.2%) and lignin (61.4%) degradation [29]. The dominant microorganisms in this consortium were bacteria *Joostella marina*, *Flavobacterium beibuense*, *Algoriphagus ratkowskyi*, *Pseudomonas putida*, and *Halomonas meridiana*, whereas *Sarocladium strictum* was the single fungal strain, indicating that bacteria have a major role in lignocellulose degradation under saline conditions. Gilmore et al. selected a stable native consortium for efficient lignocellulose-to-methane bioconversion via antibiotic treatment and serial cultivation, dominating by an anaerobic fungus (*Piromyces*), a bacterium (*Sphaerochaeta*) and two methanogenic archaea (*Methanosphaera* and *Methanocoprpusculum*) (Fig. 2A) [25]. Via the “top-down” strategy, our lab also obtained several lignocellulose degradation microbial communities from coastal waters of the East China Sea and revealed several previously unrecognized marine bacterial lignin-degraders, e.g., *Yangia*, *Pelagibaca*, *Salipiger*, *Celeribacter*, and *Vibrio* [30]. To date, microbial consortia with the ability to degrade lignocellulose have been acquired from forest soil, canal sediment, decaying wood, sea sediment, and coastal sea water [28, 31–34]. Moreover, these consortia commonly exhibited higher lignocellulose degradation and production of value-added products than single strain. The DM-1 consortium and the community, enriched from salt marsh soil, showed 28–64% lignin consumption [28, 29], whereas pure culture (e.g., *R. opacus* PD630 and *Pseudomonas putida* A514) showed 15–18% lignin consumption [12, 35]. In addition, the consortium, acquired by Gilmore et al., produced 0.75–1.9-fold more methane-rich biogas than a monoculture of fungi from the community [25].

Metagenomic sequencing analysis has further revealed key lignocellulose degrading genes and pathways in lignocellulose-degrading consortia. This greatly boosted the study of lignin bio-degradation, which has not been fully characterized, in contrast to the firm understanding of cellulose degradation. For instance, metagenomic sequencing analysis demonstrated that the lignin-degrading consortium (LigMet), enriched from a sugarcane farm, employed peroxidases, dye-peroxidases, laccases, carbohydrate esterases, and lignocellulosic auxiliary activities to oxidize lignin, and utilized benzoate-to-catechol degradation pathway, catechol ortho-/meta-cleavage pathway, and phthalate degradation pathway to catabolize lignin-derived aromatic compounds [36]. In addition, metagenomic and quantitative stable isotope probing experiments suggested aryl alcohol oxidase genes were the most significantly correlated with the enriched bacterial lignin degradation consortium from forest soils [21]. Interestingly, metagenomic sequencing detected that bacteria in Eastern Mediterranean Sea potentially utilized the phenylacetyl-CoA pathway for lignin degradation [34].

These “top-down” enriched microbial consortia, which contributed to lignocellulose bioconversion, have improved our understanding of microbial strains and key genes/pathways for lignocellulose degradation. However, it’s worth noting that consortia developed via this strategy are complex, and may contain thousands of fungal, bacterial, and archaeal members. Such complexity makes it difficult to untangle the interactive network that is responsible for the conversion process. In addition, not all effective strains from the native enrichments can be cultured in the laboratory, and further, may cause instability of community composition and function in these natural communities during sequential enrichment cultivation.

### “Bottom-up” co-cultures

To address this issue of complexity and instability, various studies about “bottom-up” route have been reported to construct consortia with a limited number of culturable strains for more efficient lignocellulose conversion. First, “bottom-up” reconstruction can be guided by “top-down” enrichment. As stated earlier, Gilmore et al. acquired a native consortium for lignocellulose-to-methane bioconversion, via the “top-down” enrichment method (Fig. 2A) [25]. The microbial composition and potential syntrophic mechanisms, which were identified by high-throughput sequencing, guided the design of a minimal consortium (Fig. 2B) [25]. This resulted in a minimal, but effective community, including fungi *Neocallimastix californiae* and *Anaeromyces robustus* with the methanogen *Methanobacterium bryantii*. In addition, Díaz-García et al. developed a combined “top-down” enrichment strategy, coupling dilution-to-stimulation and dilution-to-extinction, to build a minimal and effective lignocellulolytic microbial consortium (MELMC), where two bacterial species (*Pseudomonas* sp. and *Paenibacillus* sp.) are highly abundant (> 99%) [27]. Second, a method for efficient combination of diverse microbial strains have been reported to construct communities for lignocellulose degradation. Co-cultivation of microorganisms with complementary activities is a simple, yet effective strategy to construct efficient consortia [37, 38]. For instance, co-cultures of cellulolytic *Clostridium thermocellum* with ethanol-producing *Thermoanaerobacter* strain significantly improved ethanol production in the defined medium with 1% of cellulose, ~ 65 mM by co-cultures vs < 13 mM by C. *thermocellum* mono-culture [39]. However, microbial interactions are varied and complicated. Thus, a large number of experiments are required to examine the various effects of different microbial combinations. To overcome this limitation of intensive labor, Hu et al. reported a method for screening possible microbial interactions based on as many strains as possible, but with as few experiments as possible (Fig. 2C) [40]. In this strategy, multiple cellulolytic fungal strains were, firstly, combined in various ways to develop a synergistic fungal community, which exhibited high lignocellulosic enzyme activity. Next, a screened microbial community, which included thousands of strains, was introduced as an additional “member” of this community. Direct microbial interactions and the key strains in such interactions were identified by measuring lignocellulolytic enzyme activity and high-throughput sequencing technology [40]. As a result, two fungal (*Trichoderma* and *Aspergillus*) and 16 bacterial strains (*Bacillus*, *Enterococcus*, *Lactococcus*, *Acinetobacter*, and *Pseudomonas*) were designated as the final members of a synergistic microbial consortium with improved capacity for lignocellulolytic enzyme production (Fig. 2C). Among them, the β-glucosidase activity of the microbial community was 197% higher than that of the signal fungal strain, *Trichoderma reesei* [40]. Compared to the simple combinations of a limited number of strains, this approach could identify beneficial interactions among microorganisms and further improve those interactions by optimizing the composition and structure of the microbial community. Third, a reductive-screening approach, combined with ecological strategies, was utilized to acquire a minimal active microbial consortium (Fig. 2D). Enrichment of microorganisms is performed first, followed by isolation, identification, and metabolic characterization. Subsequently, a set of strains representing these groups are used to construct minimal active microbial consortia (MAMC). Each consortium contains different species, which vary in the number of functional groups, metabolic potential, and degradation capacity (Fig. 2D). Via this reductive-screening method, Puentes-Téllez and Falcao Salles identified 45 soil bacterial strains and classified them to four functional metabolic groups [37]. Finally, they successfully developed an effective MAMC with a 96.5% degradation rate. The MAMC contained all 18 species, where *Stenotrophomonas maltophilia*, *Paenibacillus* sp., *Microbacterium* sp., *Chryseobacterium taiwanense*, and *Brevundimonas* sp. are the major lignocellulose degraders [37].

These developed microbial consortia, via either “top–bottom” enrichment or “bottom-up” combination methods, employ microbial interactions to efficiently perform the task of lignocellulose conversion. With the advances in metagenomic and transcriptomic sequencing, it’s convenient for us to understand the microbial composition, function genes, and dynamics of these communities. However, there is not yet a clear, basic mechanistic understanding of the individuals that drive the overall function and ecological principles in these communities. To understand these complex communities and further manipulate them with defined functions, we should explore microbial interactions (e.g., modes and mechanisms) and how these properties can be applied to the rational design of bottom-up synthetic microbial communities, instead of current strategies of co-culturing microorganisms via random combinations or intuition about simple metabolite exchange.

## Microbial interaction modes and molecular mechanisms

### Synergistic microbial interaction modes

Microbial interactions are believed to be central to the survival, stability, and productivity of communities. There are six different categories of pairwise interaction modes, including neutralism (0/0), commensalism (+/0), amensalism (−/0), mutualism (+/+), competition (−/−), and parasitism or predation (±) [41]. In this review, we focus on the symbiotic interactions (commensalism and mutualism) in microbial consortia, which drive members to cooperate and execute different, yet complementary tasks.

**Fig. 3:**
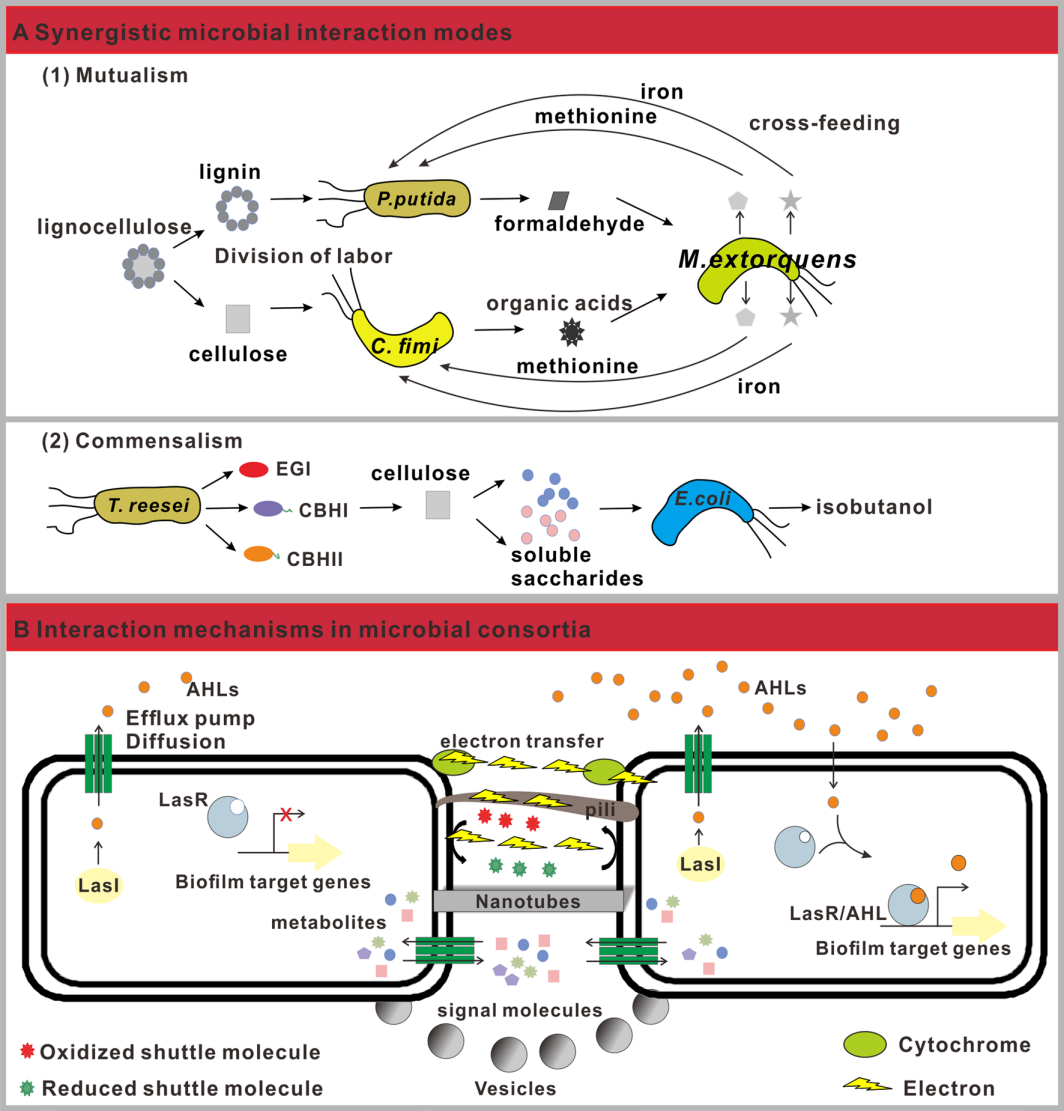
Synergistic microbial interaction modes and mechanisms. **A** Synergistic microbial interaction modes. Mutualism and commensalism are presented. CBHI, cellobiohydrolase I; CBHII, cellobiohydrolase II; EGI, endoglucanase I. Three cellulases which are produced by *T. reesei*, hydrolyze cellulose to soluble oligosaccharides. **B** Microbial interaction mechanisms. These include contact-independent and contact-based interactions. Contact-independent interactions indicate that molecules (e.g. QS signal molecules) are released by diffusion or transported by efflux pumps. Contact-based interactions suggest that molecules are exchanged by physical cell–cell contact, e.g., pili, nanotubes, vesicles. AHLs: QS signal molecules. AHL signals, which are produced by LasI family, bind LasR, thus activating it. The activated complex modulated transcription of biofilm synthesis genes. Direct electron transfer is based on membrane bound c-type cytochromes or pili, indirect electron transfer is mediated by electron shuttles

The essence of microbial synergistic interactions is the sharing and exchange of public goods. Public goods (e.g., enzymes, amino acids, vitamins and detoxification agents) are products that are costly to produce, but provide a benefit to members of a community, especially to neighbors of the producer [42–44]. For lignocellulosic degradation consortia, carbohydrate monomers and aromatic monomers, which can be directedly utilized by the members, are representative public goods. The sharing of public goods creates an opportunity for cooperative interactions, one of which is mutualism (+/+). It is the win–win relationship of symbiotic associations, in which each member derives benefits from one another (Fig. 3A) [41, 45]. Metabolic division of labor (DOL) is a typical mutualistic interaction, where distinct populations perform different, but complementary, metabolic tasks to diminish the metabolic burden on each population [46]. This was demonstrated in the synthetic lignocellulose-degrading microbial community, including *Pseudomonas putida*, *Cellulomonas fimi*, and *Methylorubrum extorquens* [47]. *P. putida* is a lignin degrader, while *C. fimi* is a cellulose degrader and these organisms work together to degrade lignocellulose (Fig. 3A). Moreover, metabolic cross-feeding is also a well-known DOL, where different groups exchange costly metabolites to the benefit of both interacting partners. In this consortium, *P. putida* produced formaldehyde and *C. fimi* generated organic acids to support the growth of *M*. *extorquens*. In turn, *M*. *extorquens* supplied methionine and iron to *P. putida* and *C. fimi* (Fig. 3A) [47].

With the succession of microbial communities, public goods could be exploited by selfish cheaters, which generates another synergistic interaction, commensalism (+/0). In this relationship one partner acquires benefits from the other, while the other one is in unaffected (neither harmful nor beneficial) [45]. In lignocellulose degrading microbial community, these opportunistic organisms are termed “sugar cheaters” [26]. Minty et al. constructed a fungal-bacterial consortia (TrEc), where *Trichoderma reesei* secreted cellulase enzymes to hydrolyze lignocellulose into soluble saccharides, as the public goods, and *Escherichia coli* metabolized soluble saccharides into isobutanol [48]. In this consortia, *T. reesei* acted as a cooperator (+) by secreting metabolically expensive cellulase, while *E. coli* was a “sugar cheater” (0), which directly utilized the saccharides without bearing the burden of cellulase production (Fig. 3A). Together, the two types of synergistic interactions enable communities to better accomplish tasks that are more metabolically intensive than pure cultures could.

### Interaction mechanisms in microbial consortia

To achieve the above stated interactions, microorganisms mostly employ two major types of interaction mechanisms, contact-independent and contact-based interactions. Contact-independent interactions rely on the release and sensing of chemical molecules (Fig. 3B) [49]. Quorum-Sensing (QS) is one of the major means to program cell–cell contact-independent communication [50]. Bacteria produce and release small hormone-like signal molecules, termed autoinducers (AI), into the environment, while neighboring microbial cells detect and respond to these molecules to alter gene expression and synchronize community-wide behavior [51]. QS mediate intra- and inter-species communication, control many microbial physiological behaviors (e.g., competence, symbiotic interactions, motility, and biofilm formation) as well as coordinate population-level behavior, thus playing a key role in lignocellulose conversion (Fig. 3B) [23, 50]. For instance, QS regulates biofilm synthesis, which can be utilized to improve lignocellulose conversion [52, 53]. On one hand, the concentration of cell-associated hydrolytic enzymes at the biofilm-substrate interface can be increased to improve saccharification [53, 54]. On the other hand, symbiotic biofilms, which consists of aerobic fungi (e.g., *T. reesei*) and anaerobic bacteria (e.g., *L. pentosus*), is an ideal strategy for simultaneous (hemi) cellulose hydrolysis and products (e.g., lactic acid) generation [55, 56]. Beyond QS signal molecules, a large repertoire of metabolites are actively or passively diffused among microorganisms to establish cell–cell interactions, including small (e.g., H_2_, CH_4_, CO_2_, and lactate) and large molecules (peptides and proteins) (Fig. 3B) [49]. In addition, electron exchanges also occur between bacteria via contact-independent interactions (Fig. 3B) [57]. Such extracellular electron transfer, via soluble electron shuttles, fuels cellulose hydrolysis and lignin oxidation [58, 59].

Meanwhile, microorganisms have evolved contact-dependent delivery systems (e.g., pili, nanotubes, outer membrane vesicles and channels) to transfer metabolites, including amino acids, proteins, DNA, and other small molecules (Fig. 3B). In addition to these macromolecules, intraspecies and interspecies electrons can be transferred via direct contact-based interactions, mediated by outer membrane cytochromes and conductive pili [45, 60]. For instance, *Staphylococcus aureus* can oxidize cellulose and generate the electricity in MFC (microbial fuel cells), via directed electron transfer [61, 62].

Contact-independent communication allows molecules to reach many neighboring cells, as opposed to only one cell at a time (e.g., cell-to-cell contact). Therefore, it enables them to serve as nutrients or cues to nearby cells, and, in effect, lead to behavior similar to that of a multicellular entity [49]. However, unintended third parties may intercept the signal or catabolize the metabolite, causing the exchanged molecules to be degraded or lost during the diffusion process [63]. In contrast, contact-dependent communication systems can overcome this issue, although they cannot simultaneously connect to multiple neighboring cells. Such synergistic microbial interaction modes and mechanisms provide insights to the following “bottom-up” design of synthetic consortia for lignocellulosic bioconversion.

## Synthetic ecology provides an opportunity to “bottom-up” design synthetic microbial consortia for efficient lignocellulose bioconversion

With the rapid development of high-throughput sequencing, the top-down study of microbial communities efficiently reveals their composition and function, further guiding us to obtain minimal, yet efficient, synthetic consortia. However, the contributions of individual members and the highly inter-connected networks of metabolic and ecological interactions are still unclear, which greatly restricts development of the “bottom-up” study of microbial consortia to enhance lignocellulose bioconversion. Synthetic ecology, which is the integration of synthetic biology, ecology, and computational biology, presents a feasibility to rationally engineer microorganisms at the population-scale. There are three important factors that should be considered for “bottom-up” synthetic ecology study of microbial consortia. These factors and corresponding methods/tools are discussed below.

### Current cooperation strategies and computational approaches to design cooperation

**Fig. 4:**
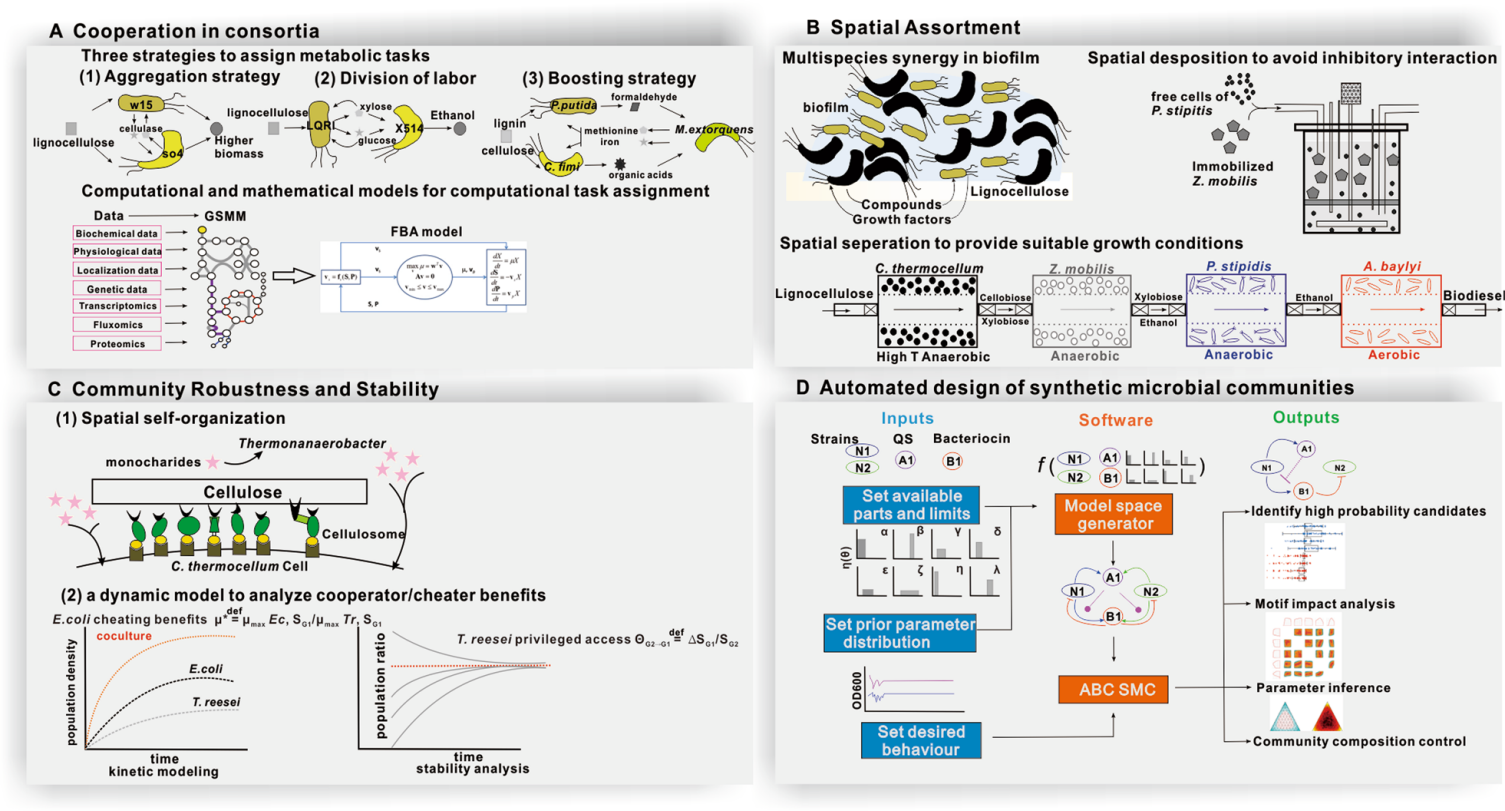
Key factors of design and engineering microbial communities for lignocellulosic conversion. **A** Microbial cooperation can be designed to perform the task of lignocellulose bioconversion. Computational and mathematical models are effective tools for genome-wide explore task assignment. **B** Spatial assortment contributes to microbial cooperation to degrade lignocellulose. Biofilms, formed by degraders or producers, provide an interface in which multiple species synergize for lignocellulose conversion. In addition, modified bioreactors can achieve spatial separation for microbial members which have different growth conditions. **C** Robustness and stability of communities can be maintained by spatial self-organization and regulation of relative cooperator/cheater benefits. **D** Overview of pipeline for the automated design of synthetic microbial communities [95]. It provides a promising avenue for the effective and easy design of microbial communities for lignocellulosic conversion

Cooperation in consortia is the first key factor to achieve efficient and stable bioconversion (Fig. 4A). So far, synthetic microbial consortia mainly employ three strategies to engineer intercellular cooperation. The first is an aggregation strategy (Fig. 4A), where every member in the consortium can individually perform the task, but each one underperforms the community. Cortes-Tolalpa et al. employed this strategy to develop an efficient microbial community for lignocellulosic biomass degradation. They utilized *Citrobacter freundii* and *Sphingobacterium multivorum*, both of which are lignocellulosic degraders. *S. multivorum* w15 secreted extracellular enzymes (e.g., cellobiohydrolases and β-xylosidases) to generate public goods, while *C. freundii* so4 provided redox power and metabolic intermediates for *S. multivorum* w15 [64]. Hence, compared to each pure culture, this coculture showed significantly increased enzymatic activities (e.g., cellobiohydrolases, mannosidases and xylosidases) and biomass (18.2-fold). The second strategy is a metabolic division of labor (DOL) (Fig. 4A). He et al. co-cultured cellulolytic *C*. *thermocellum* LQR1 and ethanol-producing *Thermoanaerobacter* sp. X514 to enhance cellulosic bioethanol fermentation [39]. LQR1 secreted the cellulosome to hydrolyze cellulose and produce soluble monosaccharides/oligosaccharides for the growth of LQR1 and X514, while X514 produced ethanol. They worked together to achieve cellulosic ethanol production. The third strategy is a boosting method (Fig. 4A). Via a cross-feeding interaction, members without the defined functions (e.g., lignocellulose degradation and product biosynthesis) contribute to the positive performances of other members with those functions. As stated previously, in the three-species lignocellulose-degrading consortia, *P. putida* and *C. fimi* were lignocellulose degraders, while *M. extorquens* removed a toxic burden and fed back iron and methionine to the degraders for improvement of lignocellulose degradation (Fig. 3A) [47].

The essence of design cooperation is understanding how metabolic tasks can be rationally allotted to individual members to achieve a desirable productive manner at a community-level. However, microorganisms have complex cross-feeding strategies for multiple metabolites, either simultaneously, or in an environment-dependent manner [65, 66], undoubtedly leading to the manual design of cooperation to be very challenging. Mathematical models and computational approaches in synthetic ecology have been developed to characterize microbial interactions and complex metabolic networks at the genome level. Minty et al. developed a comprehensive ordinary differential equation (ODE) modeling framework for the TrEc consortium (consisting of cellulolytic specialist *T. reesei* and isobutanol producer *E. coli*), including growth kinetic model, cellulase secretion model and saccharide uptake model for *T. reesei*, as well as growth model, saccharide uptake model and isobutanol production for *E. coli* [48]. Fifty parameters and variables for the concentration of microbial biomass, enzymes, isobutanol, soluble oligosaccharides, and each possible cellulose polysaccharide were introduced into the models to capture salient features of the TrEc consortium. It suggested that competition between *T. reesei* and *E. coli* for soluble saccharides was a key ecological interaction to drive community behavior, indicating an inherent tradeoff between cellulose hydrolysis rate and isobutanol yield [48]. Through this experimentally validated mathematical modeling framework, key parameters (e.g., growth kinetics, substrate uptake kinetics, and population ratio), which controlled consortium behavior, were identified and optimized. As a result, isobutanol yields of this consortium increased to 62% of theoretical maximum, reaching titers of 1.88 g/L [48]. In addition, in silico genome-scale metabolic models (GSMM) have been constructed to combine experimental and omics data sets in a systematic manner to understand complex microbial metabolism [67–69]. GSMM are constructed in a bottom-up fashion based on genomic and bibliomic data, thus representing the biochemical, genetic, and genomic (BiGG) knowledge for the target organism (Fig. 4A) [70]. Based on GSMM, a variety of flux balance analyses (FBA) were performed, including parsimonious flux balance, dynamic flux balance, and flux variability analysis [65, 68, 71, 72]. Such flux analysis can determine how a cell should optimally allocate nutrients so that the growth rate is maximized [73, 74]. Moreover, it can be extended to microbial communities to study metabolic interactions and predict community-wide behaviors [75]. Hanly et al. employed dynamic flux balance modeling to improve the consumption of glucose/xylose mixtures (the main enzymatic products of cellulose degradation) in *Saccharomyces cerevisiae* and *E. coli* co-cultures [76]. In this consortium, *S. cerevisiae* consumes glucose, while *E. coli* strain ZSC113 catabolizes xylose. Inhibitory interactions were observed: *S. cerevisiae* metabolizes some compounds, which are toxic to *E.coli*, and produces ethanol that inhibits the growth of *E. coli*. Thus, the efficiency of glucose/xylose utilization was not high. The dynamic flux balance model suggested that optimization of co-culture inoculum can address such problem, as optimized inoculum can accelerate xylose uptake rates at the expense of decrease glucose uptake. As a result, the co-culture system can exhaust 16 g/L glucose and 8 g/L xylose at 9.8 h, compared to original 11.5 h batch time [76]. Additional in silico genome-scale models have been developed to simulate better programs about microbial interactions [77]. For instance, division of labor in metabolic networks (DOLMNs) were reported to explore how metabolic interdependencies between metabolically different *E. coli* strains [65]. A multiphase multi-objective dynamic genome-scale model of was constructed to provide insights into how different redox balancing was achieved among *Saccharomyces* strains [78]. These models offer a systematic approach to expand on the engineering intercellular cooperation for lignocellulosic bioconversion.

### Spatiotemporal assortment and related biotechnology

Spatial and temporal assortment should be considered to promote cooperation, although microbial communities are generally cultured in well-mixed systems. Various microbial spatial and temporal segregations exist in nature. For example, aerobic and anaerobic microbial populations are separated by oxic/anoxic niches [22], whereas biofilms provide structured microenvironments [53, 56, 79, 80]. Such spatial organization of microbes generates locally heterogeneous subpopulations, which can acquire different resources, and further enhance local interactions. Hence, considerations of spatial disposition should improve system robustness and productivity (Fig. 4B). Biofilm-based separation shed light on enhancement of lignocellulosic bioconversion. For a cellulose degrader, biofilms can not only strengthen physical contact between microbial cells and cellulose substrate, but also enhance concentration of cellulases at the biofilm-substrate interface to promote hydrolysis rates, e.g., 70% higher cellulase activity by *Aspergillus niger* biofilm, compared to suspended *A. niger* culture [53, 81]. For a product producer, biofilm also increased biochemical production, due to the increment of surface area for mass transfer and cell resistance against adverse environment [53, 56]. Higher ethanol concentration (5.16 g/L and 5.33 g/L) was produced from rice husk hydrolysate (RHH) via *Scheffersomyces stipitis* in a biofilm reactor (BR) and continues plastic composite support (PCS)-BR, respectively [82, 83]. For microbial consortia, biofilm further provides a layered microbial structure, in which multispecies synergize for simultaneous delignification and saccharification or co-fermentation of pentose and hexose (Fig. 4B) [52]. The biofilm of *T. reesei*, which was co-cultured with *S. stipitis* (cellulose degrader) and *S. cerevisiae* (ethanol producer), can contribute to ethanol production (9.8 g/L) from the undetoxified dilute acid pretreated wheat straw [84]. Moreover, biofilm mediated spatial separation provides a strategy to resolve the physiological incompatibility of microorganisms. In the cellulose to lactate conversion system, aerobic *T. reesei*, which secretes cellulase to hydrolyze cellulose, formed biofilm on the surface of tubular membrane. As a result, biofilm consumed oxygen and generated anaerobic microenvironment for the growth of anaerobic *Lactobacillus pentosus* for lactate production (34.7 g/L) [53].

Additional positive effect of spatial separation was achieved via modified bioreactors (Fig. 4B). Fu et al. used beads in a modified fermenter to achieve spatial separation between the bacterium *Zymomonas mobilis* and the yeast *Pichia stipitis* for efficient lignocellulosic ethanol production (Fig. 4B) [85]. *Z. mobilis* can ferment glucose, while *P. stipitis* can ferment xylose. However, viable *Z. mobilis* inhibited xylose fermentation by *P. stipitis*, due to the oxygen deprivation by *Z. mobilis*. Thus, Fu et al. developed a novel co-culture process, where immobilized *Z. mobilis* beads were used, instead of free *Z. mobilis* in the co-culture, to decrease the interaction between these two strains (Fig. 4B). As a result, the efficiency of the xylose fermentation was significantly enhanced. Moreover, ethanol yield achieved 0.477 g/g, which is more than 96% of the theoretical value. In addition, in a lignocellulosic-to-biodiesel production system, *C. thermocellum* anaerobically hydrolyzes lignocellulose into hexose and pentose [86]. Next, *Z. mobilis* and *P. stipitis* anaerobically ferment hexose and pentose to ethanol, whereas downstream *A. baylyi* aerobically converts ethanol to biodiesel. The four strains were cultured in different bioreactors to maintain the most suitable growth conditions (e.g., temperature and oxygen) for each strain and avoid cross-inhibition, while hollow fiber bridges connected the bioreactors to exchange intermediate products (Fig. 4B). It is worth mentioning that microfluidic and microwell platform also can efficiently control spatial structure and metabolite communication at micron scale [22, 87]. Such high spatial resolution techniques should offer an attractive alternative for microbial spatial assortment to improve lignocellulosic bioconversion processes, especially those that are upstream.

In addition to spatial segregation, temporal separation also could be used to resolve physiological or metabolic incompatibilities. As mentioned previously, *Z. mobilis* and *P. stipitis* have cross-inhibition. Fu et al. also developed the sequential culture strategy to boost glucose/xylose co-fermentation. Free *Z. mobilis* was first inoculated to consume glucose and subsequently free *P. stipitis* was inoculated to ferment xylose when *Z. mobilis* was inactivated [85]. Moreover, sequential activities (e.g., gene expression) in distinct growth phases can achieve temporal separation, although synthetic consortia have yet to be specifically designed with this concept [22]. The gene circuits, which have feedback loop architecture, could have temporally coordinated oscillations across the population, through intercellular communication mediated by chemical molecules. Well-known quorum sensing and quenching, which produce chemicals to sequential turn on/off gene expression, is an ideal circuit to design temporal separation of lignocellulosic conversion microbial communities in the future [88, 89].

### Sub-population ratio control for community robustness and stability

Stability of microbial consortia is essential, yet challenging. For microbial consortia in nature, the environment causes community change, where consortia may experience environmental fluctuations and exposure to competitive species. Moreover, succession of microbial consortia changes the community due to genome evolution and horizontal gene transfer [90]. For synthetic microbial communities, sub-population ratio, especially ratio of cooperator-cheater, is the important factor that interferes with the community stability. Cooperators produce enzymes, which catalyze substrates, and have a lower growth rate than that of cheaters, as they consume more energy to synthesize enzymes. In case that enzymatic products are equally shared by cooperators and cheaters. Cheaters would dominate the system and cause collapse of the microbial community. In contrast, “Snowdrift game” suggests that cheaters and cooperators can stably coexist when cooperators can acquire enough net benefits in systems [91]. Two strategies have been utilized to protect the interests of cooperator in lignocellulose bioconversion microbial consortia. (i) Spatial self-organization contributes to cooperation (Fig. 4C) [92]. Positive assortment can enable cooperation without being taken over by cheaters. Take cellulosomes for instance, previous studies demonstrated that the cell-bound cellulosome had 2.7–4.7-folds higher hydrolysis rate than their free counterparts, containing the same cellulases [93]. This should be the result of substantially higher substrate retention at cell surface and enhancement of the local hydrolysate concentration. Thus, in the co-culture system of *C. thermocellum* (a cooperator) and *Thermoanaerobacter* (a cheater), the cooperation of cellulose-to-ethanol conversion was stable, since higher rewards (hydrolysates) are received by *C. thermocellum* and favor its growth. Such spatial self-organization of cellulases in the cooperator contributes to co-exist of the two strains. (ii) Regulation of relative cooperator/cheater benefits is another strategy to resist cheaters and maintain cooperation (Fig. 4C). Minty et al. constructed a dynamic model to perform a stability analysis on a simplified version of the TrEc consortium model for cellulosic isobutanol production. Their analysis suggested that ecological parameters, including growth kinetics, substrate uptake kinetics and carbon flow partition, determined the tradeoff between cellulose hydrolysis rate and product yields. Thus, manipulation of these parameters can control the relative cooperation/cheating benefits to maintain the stability of the TrEc consortium (*T. reesei*/*E. coli*) [48].

Moreover, precise population control is also a desirable method to maintain the stability of communities. Genetic parts, e.g., QS systems and bacteriocins, are useful elements to manipulate the growth rate or subpopulation fitness [45, 94, 95]. These elements should provide genetic tools to further construct synthetic microbial community for lignocellulose bioconversion.

### Automated and computational design of synthetic microbial consortia

With the increasing number of potential types of engineered interactions and community members, manual judgement of these considerations is cumbersome and inefficient, representing a major hurdle in synthetic microbial community. Automated design of synthetic microbial communities has been recently developed to counter this hurdle [95]. This methodology enables researchers to automatically build up computational circuit design, including engineer metabolic interdependencies mutualism and employ QS/bacteriocins to control community stability. Hence, model selection can be computationally performed to identify the most promising designs [95] (Fig. 4D). This framework of design-build-test cycle can generate impactful rules and heuristics for building a robust and stable synthetic community with a desired behavior, laying foundation to further design lignocellulose bioconversion consortia.

## Conclusion and perspectives

Bottom-up synthetic ecology study of microbial communities, which precisely consider the properties of individual functions, interactions, communities, and applications, yield a better understanding for diversity, interactions, and dynamics at the systems level. Hence, it presents an exciting opportunity to rationally engineer microbial consortia for lignocellulose bioconversion. However, we still lack the critical knowledge and technology to effectively characterize the contributions of individual members, reveal the highly inter-connected networks of metabolic and ecological interactions, as well as perform genetic manipulation at the systems-level. There are two major barriers, which need to be addressed in the next step. First, it’s difficult to gain a fine-scale understanding of functions of individual microbial species and a subsequent large-scale understanding of ecosystem function. So far, this has mainly relied on genome-scale and computational modeling approaches. The qualities of these in silico models greatly depend on the accuracy and completeness of information about metabolic networks and biochemical roles. For most microbial species in natural environments, such information is missing and requires significant time and effort to accumulate. Thus, it is restricted to constructed synthetic communities which are comprised a few (e.g., 2–3 strains) well-characterized species. Development of automated approaches for metabolic reconstructions of unmodelled strains would alleviate this bottleneck. Alternatively, it would be desirable for the extension of computational approaches to design communities which contain novel species without genome-scale metabolic reconstructions. Second, it’s challenging to genetically manipulate microbial communities with a wide range of specificities and magnitudes. So far, genetic engineering is focused at the level individual strains. In contrast to breakthroughs in high-throughput sequencing to obtain molecular information of microbiomes, microbiome genetic modulation has not seen widespread success. With the recent revolution in genome engineering toolboxes [96], genetic modification of a community’s metagenomic content provides an avenue to achieve the desired manipulations [97]. Moreover, directly manipulating in situ bacterial communities in an open and changing environment could offer an exciting route for lignocellulose bioconversion.

## Data Availability

Not applicable.

## Abbreviations

PHA: Polyhydroxyalkanoate
GHs: Glycoside hydrolases
DypBs: B type dye-decolorizing peroxidases
LigMet: Lignin-degrading consortium
MELMC: Minimal and effective lignocellulolytic microbial consortium
MAMC: Minimal active microbial consortia
QS: Quorum-Sensing
AI: Autoinducers
MFC: Microbial fuel cells
ODE: Ordinary differential equation
GSMM: Genome-scale metabolic models
BiGG: Biochemical, genetic, and genomic knowledge
FBA: Flux balance analyses
DOLMNs: Division of labor in metabolic networks
